# Metabolic syndrome among people living with HIV on antiretroviral therapy in Mwanza, Tanzania

**DOI:** 10.1186/s12902-023-01340-3

**Published:** 2023-04-21

**Authors:** Evangelista Malindisa, Emmanuel Balandya, Marina Njelekela, Benson R. Kidenya, Filbert Francis, Blandina T. Mmbaga, Haruna Dika, Eligius Lyamuya, Bruno Sunguya, John Bartlett, George PrayGod

**Affiliations:** 1 Department of Physiology, Catholic University of Health and Allied Sciences Bugando, P.O. Box 1464, Mwanza, Tanzania; 2 Muhimbili University of Health and Allied Sciences, P.O. Box 65001, Dar Es Salaam, Tanzania; 3 Deloitte Consulting Limited, P.O. Box 1559, Dar Es Salaam, Tanzania; 4 Department of Biochemistry and Molecular Biology, Catholic University of Health and Allied Sciences Bugando, P.O. Box 1464, Mwanza, Tanzania; 5 Kilimanjaro Christian Medical University College, P.O. Box 2240, Moshi, Tanzania; 6 Mwanza Research Centre, National Institute for Medical Research, P.O. Box 1462, Mwanza, Tanzania; 7 Duke Global Health Institute, Duke University, Durham, NC USA; 8 Tanga Research Centre, National Institute for Medical Research, P.O. Box 5004, Tanga, Tanzania

**Keywords:** MetS, HIV, Antiretroviral therapy

## Abstract

**Background:**

HIV and antiretroviral drugs, particularly protease inhibitors and nucleoside reverse transcriptase inhibitors, may increase the risk of Metabolic Syndrome (MetS) among people living with HIV (PLHIV). However, following the introduction of better drugs like dolutegravir, data on the burden of MetS are limited. This study aimed to assess the prevalence of MetS and associated factors among PLHIV on antiretroviral therapy (ART) in Tanzania.

**Methods:**

This was a cross-sectional study among PLHIV aged ≥ 18 years on antiretroviral therapy for ≥ 1 year at Bugando Medical Centre in Mwanza conducted in 2020. Demographic and healthy-lifestyle-related non-communicable disease risk factors data were collected. Additionally, data on lipid profile, blood glucose, blood pressure, and waist circumference were collected for analysis of MetS according to the International Diabetes Federation criteria. Factors associated with MetS were assessed using logistic regression. A *P* ≤ 0.05 was considered statistically significant.

**Results:**

Data for 223 participants were analyzed. The mean (SD) age was 44 (± 12) years and 79.8% (178) were females. A majority 78% (174) were on a tenofovir, lamivudine,and dolutegravir regimen. About 12.1% (27) were either current or past smokers, 45.3% (101) were past alcohol drinkers, 22.9% (51) were current drinkers, 12.1% (27) reported taking ≥ 5 servings of vegetables and fruits per day and 5.8% (13) were physically inactive. The prevalence of MetS was 22.9%. The only factors that were associated with Mets were fat mass index and adequate intake of vegetables and fruits, (adjusted odds ratio (aOR) 2.9, 95% CI 1.0, 7.9, *P =* 0.04) and (aOR1.2, 95% CI 1.0, 1.3, *P =* 0.02), respectively).

**Conclusion:**

The prevalence of MetS remains high among PLHIV. Adiposity and adequate fruit and vegetable intake increased the risk. The introduction of new ART regimens shows no effect on MetS prevalence. Research is needed to understand how lifestyle changes could reduce MetS in PLHIV.

**Supplementary Information:**

The online version contains supplementary material available at 10.1186/s12902-023-01340-3.

## Background

UNAIDS reports that HIV-related mortality decreased between 2010 and 2020 due to the widespread use of antiretroviral therapy (ART) [1]. The decrease is due to ART effects on viral load suppression, body immunity improvement as well a reduction in the incidence of opportunistic infections [2]. Despite these beneficial effects, there is evidence that ART may increase the risk of non-communicable disease (NCD) co-morbidities [3–7]. Studies have shown that ART regimens, particularly those containing protease inhibitors (PIs) and nucleoside reverse transcriptase inhibitors (tNRTIs) [6, 8] could lead to HIV-associated lipodystrophy, dyslipidemia,and insulin resistance, thus increasing the risk of metabolic syndrome (MetS) [9]. MetS, previously described by Reaven as syndrome X, is a collection of closely related cardiovascular risk factors, including hypertension, insulin resistance, obesity, and dyslipidemia, with abdominal obesity and insulin resistance being the core manifestation [10–12]. MetS is associated with an increased risk of diabetes mellitus and cardiovascular diseases than its components [13]. Therefore, diagnosing MetS early could help prioritize interventions to reduce the risk of these cardio-metabolic diseases and improve the long-term health of people living with HIV (PLHIV) [13].

Although the use of PIs and tNRTIs is phasing out in sub-Saharan Africa [14], it is still important to explore the burden of MetS and its associated factors. There is limited literature on the burden of MetS in the era of newer and better ART drugs such as dolutegravir. The available evidence is contradicting, with some studies showing improvement of metabolic syndrome with dolutegravir-based regimens [15] while others showing an increased risk of weight gain [16, 17] and hyperglycemia [18]. Against this background, this study aimed to explore the current prevalence of MetS among PLHIV on ART, as well as factors associated with increased risk of MetS in this population.

## Methods

### Study design and setting

This was a cross-sectional study conducted from June to August 2020 among adults PLHIV on ART for ≥ 1 year attending Bugando Medical Centre (BMC) HIV clinic in Mwanza, northwestern Tanzania. BMC is the zonal hospital for the Lake Victoria Zone in northwest Tanzania, serving a population of approximately 15 million. The HIV prevalence in the Lake Zone is 6.5%, higher than the national average of 4.9% [19]. At the time of the study, Bugando HIV clinic had more than 15,000 HIV-infected patients enrolled with over 5700 on ART [20].

### Sample size and sampling

Based on a previous studyconducted in Mwanza in 2016, the prevalence of MetS among PLHIV was 21.7% [21]. To estimate the prevalence in the current study with a precision of 5% and 80% power we needed to recruit a minimum of 261 patients.Using Microsoft excel random sampling function, participants were randomly selected out of 5700 patients who were in care at BMC during the time of recruitment and had been on ART for ≥ 1 year. Participants were contacted by phone and introduced to the study and those not reachable were excluded. Information on study objectives, procedures including a requirement to have an overnight fast for at least 8 h, risks, and benefits were communicated to potential participants before consenting. If an individual agreed to participate in the study, an appointment was scheduled for them to attend the research clinic based on their availability, between 08:00 h to 10:00 h to minimize the possibility of breaking the fast before enrollment. Upon arrival, participants were re-introduced to the study and consented. Those who gave their written consent were enrolled in the study.

### Data collection and measurements

Using structured interviews, information on demography, education level, employment, religion, and marital status were collected. Data on possession of assets (residential house, electric or gas cooker, bicycle, motorcycle, car, sewing machine, radio, television, air-conditioning, mobile phone, animals, chicken, boat, and any rented property), source of water for domestic use and type of toilet used were collected and used to compute socioeconomic status using principal component analysis (PCA) [22], which was then grouped in tertiles (i.e. lower, middle and upper). The World Health Organization (WHO) Global Physical Activity Questionnaire (GPAQ) was used to collect reported data on the level of physical activity [23]. Total physical activity was computed to metabolic equivalents of tasks (MET) in minutes per week and categorized as an adequate level if MET was ≥ 600 as recommended by WHO [24]. Smoking status was elicited and grouped as never smoked and ever smoked (including past smokers (who quit smoking for > 1 year) and current smokers (smoking in the past year)). Alcohol consumption was grouped as never consumed, past consumption (quit intake for > 1 year), and current consumption (consuming within the past 1 year). Fruits and vegetables intake was assessed based on consumption history in the past 7 days. To facilitate data collection, we prepared a list of commonly available fruits and vegetables in Mwanza and provided a container for participants to estimate the portion of fruits and vegetables they were taking on days they reported to be taking fruits or/and vegetables. Using this information, a trained research assistant converted the quantities ingested into servings; with one serving weighing approximately 80 g. Based on WHO recommendations, we regarded an intake of ≥ 5 servings per day as being adequate [25]. ART cards were used to obtain information on ART regimens and HIV treatment duration.

Anthropometric measurements were assessed by trained research assistants using standardized methods [26]. Bodyweight was determined to the nearest 0.1 kg using a digital scale (Seca, Germany) and height was measured to the nearest 0.1 cm using a stadiometer fixed to the clinic wall (Seca, Germany). Waist and hip circumferences were measured using non-stretchable tape (to 1 mm). All measurements were taken in triplicate and means were used during the analysis. Waist circumference was used to assess abdominal obesity which is one of the International Diabetes Federation (IDF) MetS criteria; where waist circumference of ≥ 80 cm for females and ≥ 94 cm for males were defined as abdominal obesity [27]. Participants underwent bioelectrical impedance analysis (BIA) to estimate fat mass (kg) using a body composition analyzer (Tanita BC418, Tokyo, Japan). To adjust for height, the fat mass index (FMI) was computed as fat mass (kg) divided by height^2^ (m).

Blood pressure was measured using a standardized protocol. Participants were asked to rest for at least 5 min, and then three serial measurements of BP were taken one minute apart, using a digital blood pressure monitor sphygmomanometer (CH-432B, Citizen Systems Japan Co Ltd) with subjects in the sitting position. Having an average systolic blood pressure of ≥ 130 mmHg and/or average diastolic blood pressure of ≥ 85 mmHg was included in the index defining MetS [28].

Fasting venous blood samples were recollected and used for the analysis of total serum cholesterol, high-density lipoproteins (HDL), low-density lipoproteins (LDL), and triglycerides using a chemistry analyzer (ERBA XL, S.R.O Mannheim, Germany). Total cholesterol > 5.2 mmol/l, triglycerides > 1.7mmol/l, and HDL < 1.03 mmol/l in males and < 1.29 mmol/l in females and LDL > 3.3mmol/l were considered impaired. HDL and triglycerides results were used in the index defining MetS [27]. Participants were contacted one day before the clinic visit and instructed to come fasting. Upon arrival and before glucose testing, participants were asked if they had fasted for at least 8 h before visiting the clinic. Those on fast were requested to provide venous blood for fasting blood glucose (FBG) analysis using a Hemocue machine (Hemocue 201 RT, Hemocue AB, Angelholm, Sweden). FBG 6.1 to 7.0 mmol/l was impaired fasting glucose while FBG > 7.0 mmol/l was diabetes.

### MetS definition

MetS was the main outcome measured and was defined as the presence of central obesity plus any two of the following: triglycerides ≥ 1.7 mmol/l or specific treatment for this lipid abnormality, HDL < 1.03 mmol/l in males and < 1.29 mmol/l in females or specific treatment for this lipid abnormality, elevated blood pressure (systolic blood pressure ≥ 130 mmHg and/or diastolic blood pressure ≥ 85mmHg) or treatment of previously diagnosed hypertension, and fasting hyperglycemia (fasting blood glucose ≥ 7.0 mmol/l) or previously diagnosed type 2 diabetes as recommended by IDF [27]. The following were the criteria in this study; **MetS1**: Presence of abdominal obesity, elevated serum triglyceride, and decreased serum HDL levels. **MetS2**: Presence of abdominal obesity, elevated serum triglyceride, and systolic blood pressure equal or more than 130 mmHg and/or diastolic blood pressure equal or more than 85 mmHg. **MetS3**: Presence of abdominal obesity, elevated serum triglyceride, and fasting blood glucose more than or equal to 7 mmol/L. **MetS4**: Presence of abdominal obesity, decreased serum HDL level, and fasting blood glucose more than or equal to 7 mmol/L. **MetS5**: Presence of abdominal obesity, decreased serum HDL level, and systolic blood pressure equal or more than 130 mmHg and/or Diastolic blood pressure equal or more than 85 mmHg. **MetS6**:Presence of abdominal obesity, systolic blood pressure equal to or more than 130 mmHg and/or diastolic blood pressure equal to or more than 85 mmHg and fasting blood glucose more than or equal to 7 mmol/L. **MetS**: Any of the 1–6 criteria [27].

### Data management and statistics

Data were entered into CSPro and analyzed in STATA version 16. Histograms and the Shapiro-Wilk test were used to assess the distribution of continuous variables. Background characteristics of the study participants are presented as medians and interquartile ranges or means and standard deviations (SDs), or percentages of categorical variables. Associations between Mets and predictor variables (age, sex, education level, marital status, employment status, smoking status, alcohol drinking, physical activity, fruit, and vegetable intake, and FMI tertiles) were investigated using logistic regression. Univariable logistic regression was conducted for all predictor variables and those whose effect sizes were significant at P < 0.1 were included in multiple logistic regressions. The odds ratio (OR) was presented with 95% CI and *P* < 0.05 indicated significant differences.

## Results

A total of 261 participants were recruited, but only 85.4% (223) had complete outcome data and were included in the analysis. The mean age of included participants was 44 (± 12) years and 79.8% (178) were females (Table 1). Among these participants, 12.1% [27] were either current or past smokers, 45.3% (101) were past alcohol drinkers, 22.9% (51) were current drinkers and only 12.1% [27] reported taking ≥ 5 servings of vegetables and fruits per day (Table 1).

**Table 1:** Background characteristics of the study participants

Characteristic	Categories	N (%)
Age	< 45 years	117 (52.5)
	≥ 45 years	106 (47.5)
Sex	Males	45 (20.2)
	Females	178 (79.8)
Education	Never	17 (7.6)
	Primary School	138 (61.9)
	Secondary and above	68 (30.5)
Employment	Unemployed/Housewife	46 (20.6)
	Self Employed	147 (65.9)
	Salary Employee	30 (13.5)
Socio-Economic Status	Lower tertile	75 (33.6)
	Middle tertile	74 (33.2)
	Upper tertile	74 (33.2)
Smoking	Never	196 (87.9)
	Ever-Smoked	27 (12.1)
Alcohol Drinking	Never	71 (31.8)
	Past-Drinker	101 (45.3)
	Current-Drinker	51 (22.9)
Vegetable and fruit intake	< 5 servings/day	196 (87.9)
	≥ 5 servings/day	27 (12.1)
Physical Activity	Active (≥ 600 MET minutes/week)	210 (94.2)
	Not Active(< 600 MET minutes/week)	13 (5.8)
Body mass index (BMI) Categories	Underweight (BMI < 18.5)	18 (8.1)
	Normal weight(BMI 18.5–24.9)	109 (48.9)
	Overweight/obese(BMI ≥ 25)	96 (43.0)
Fat mass index	-	8.5 ( SD 5.0)^3^
Antiretroviral therapy	Dolutegravir, tenofovir, lamivudine	174 (78)
	Other First-Line drugs^1^	29 (13)
	Second Line drugs^2^	20 (9)
Time since HIV diagnosis	< 10 years	69 (41.8)
	≥ 10 years	96 (58.2)

^1^Other first-line drugs (Nevirapine, zidovudine, emtricitabine, abacavir) ^2^ s line regimen (lopinavir/r and Atanazavir/r). ^3^Fat mass index has been presented as a mean with a standard deviation

**Fig. 1:**
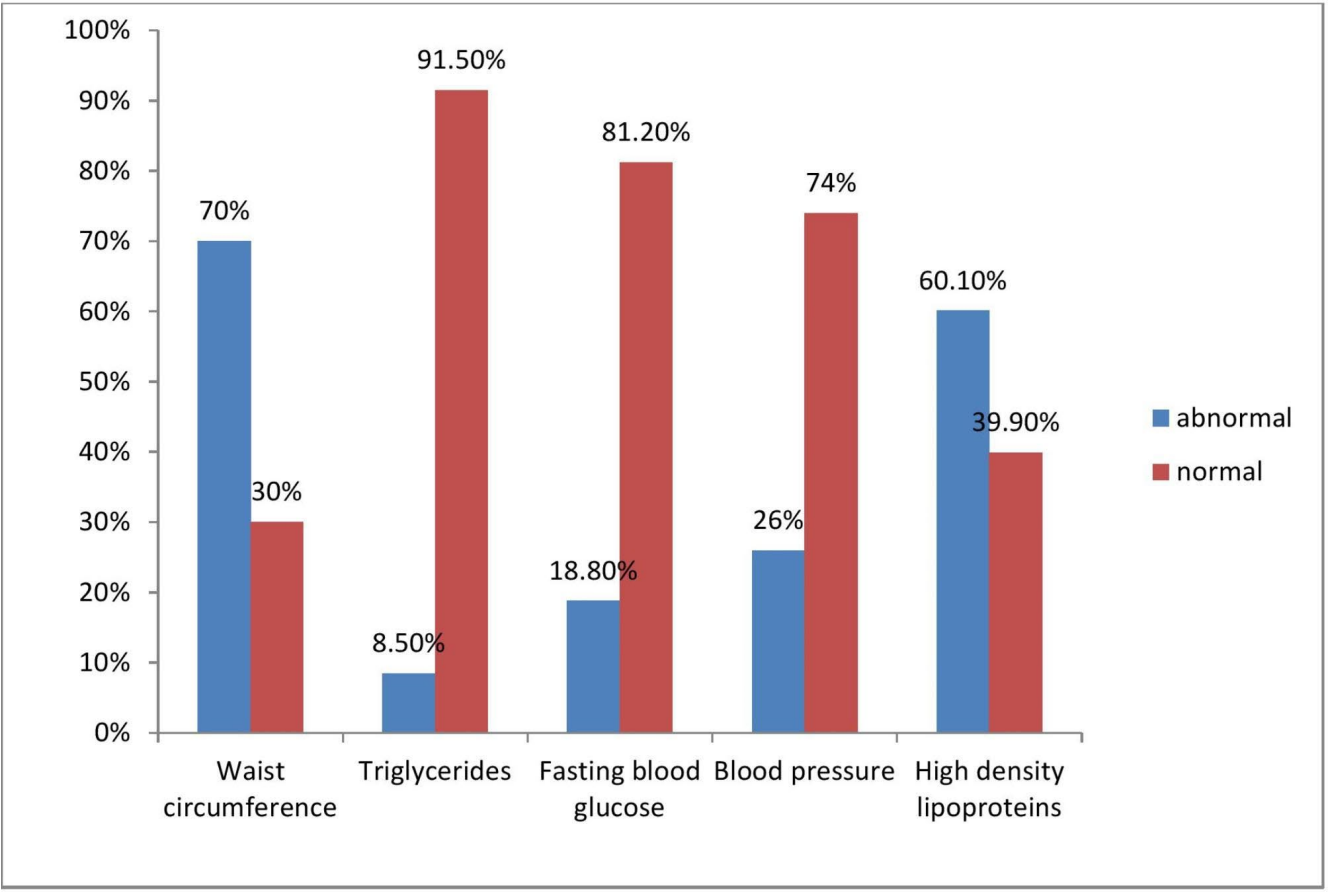
Distribution of metabolic syndrome traits among study participants [27]

Fig. 1 summarizes the proportions of each of the MetS traits of the study participants. Over two-thirds of the study participants had central obesity, 8.5% [19] had elevated serum triglycerides, 18.8% [42] had diabetes, 26% (58) had hypertension and 60.1% (134) had lowHDL cholesterol.

Of all IDF MetS criteria, the combination of abdominal obesity, low HDL levels, and hypertension accounted for the largest proportion compared to other combinations. The overall prevalence of MetS was 22.9% (51) **(Supplementary Fig. 1**).

Table 2 summarizes univariable and multivariable logistic regression analysis for the factors associated with MetS. In a multivariable model, we found that increased fat mass index and intake of adequate vegetable and fruit (≥ 5 servings per day) were associated with higher odds of having MetS ((aOR 1.2, 95% CI: 1.0, 1.3, *P =* 0.01) and (aOR 2.9, 95% CI: 1.0, 1.3, *P =* 0.04), respectively). In addition, we found that participants aged ≥ 45 years had higher odds of having MetS (OR 4.5 95% CI 2.2, 9.0, *P* = 0.001), although this was not significant in the multivariable model (aOR 2.3 95% CI 0.9, 5.9, *P* = 0.1). ART regimens and time since HIV diagnosis were not predictors.

**Table 2:** Univariable and multivariable logistic regression analysis of the factors associated with metabolic syndrome

Characteristic	Categories	Univariable Analysis		Multivariable Analysis	
		OR (95%CI)	P	Adjusted OR (95%CI)	P
Age	≥ 45 years	4.5 ( 2.2, 9.0)	**0.001**	2.3 (0.9, 5.9)	0.1
sex	Male	0.7 ( 0.3, 1.6)	0.32	2.1 (0.6, 7.6)	0.2
Education	Primary School	1.1 ( 0.3, 3.5)	0.9	-	-
	Secondary and above	0.8 ( 0.2, 2.7)	0.7		
Employment	Salary employed	0.6 (0.2, 2.1)	0.5	-	-
	Self Employed	1.0 (0.5, 2.2)	1.0		
Socio-Economic Status	Middle	0.7 ( 0.3, 1.6)	0.5		
	Upper	1.1 (0.5, 2.3)	0.8		
Smoking	Ever-Smoked	0.7 (0.3, 2.1)	0.6	-	-
Alcohol Drinking	Past-Drinker	2.4 (1.1, 5)	**0.02**	0.9 (0.3, 2.5)	0.3^3^
	Current-Drinker	0.7 (0.2, 1.9)	0.4	0.4 (0.1, 1.6)	
Vegetable/fruit eating habits	≥ 5 portions/ day	4.6 (2.0, 10.7)	**0.001**	2.9 (1.0, 7.9)	**0.04**
Physical Activity	Not active (< 600MET/week)	4.4 (1.4–13.8)	**0.01**	3.3(0.6, 18.1)	0.2
Fat mass index	-	1.1 (1.1, 1.2)	**0.001**	1.2 (1.0, 1.3)	**0.01**
ART regimen	Other First Line^1^	1.7 (0.7, 4.0)	0.2		
	s Line^2^	1.2 (0.4, 3.6)	0.7		
Time since HIV diagnosis	≥ 10 years	3.2 ( 0.3, 0.7)	**0.01**	0.6 (0.2, 1.6)	0.3

^1^Other first-line drugs (Nevirapine, zidovudine, emtricitabine, abacavir) ^2^ s line regimen (lopinavir/r and Atanazavir/r). ^3^ overall P value for alcohol drinking prediction on MetS. Age < 45, Female sex, never attended school, salary employees, no smoking, no alcohol drinking, vegetable/fruit eating habit < 5portions/day, being not active, lower fat mass index, Dolutegravir-based regimen and < 10 years since HIV diagnosis were the reference categories in the respective variables

## Discussion

The current study found the prevalence of MetS among HIV patients on ART to be 22.9%. In addition, adiposity seemed to increase the risk of MetS and we found that adequate intake of vegetables and fruits was associated with a higher burden of MetS. We also found MetS burden seemed to be higher in people aged ≥ 45 years.

The prevalence of MetS observed in this study is higher than the reported global prevalence of MetS by IDF (18%) [29]. This is similar to the previously reported prevalence in Tanzania and other countries in sub-Saharan Africa. For instance, a study conducted in urban and rural settings in Tanzania in 2015, reported a prevalence of 25.6% [30]. Another study done in the same setting as the current study between 2012 and 2013 reported a 21.3% prevalence of MetS in PLHIV on ARTs [21]. A study done in Kenya in 2016 reported a prevalence of 19.2% [31]. The similarity of proportions in the current and previous studies suggests that the change in first-line ART regimens has little to do with the MetS since the high prevalence is still observed despite changes in ART regimens and no associations have been observed between MetS and different regimens. The high prevalence of MetS is likely to be attributed to other factors including consumption of unhealthy diets, although we found a high intake of vegetables and fruits were associated with a higher rather than lower burden of MetS.

The finding that a higher intake of vegetables and fruits was not beneficial is contrary to other studies which suggest that consumption of vegetables and fruits may reduce MetS risk [32]. However, the findings of this study are similar to a recently published study in the same setting which reported that adequate intake of vegetables and fruits was associated with a higher burden of prediabetes and diabetes, although the findings did not achieve statistical significance [33]. These findings were not expected but could be due to the high glycemic index of common fruits eaten in Mwanza, such as watermelons, which in some cases maybe comparable to that of some carbohydrate-rich foods like white bread [34]. Indeed, a recent 20-year-long prospective cohort study from the Stockholm Diabetes Prevention Program, involving 6961 participants, reported an increased risk for prediabetes and diabetes among men and women with higher intakes of bananas and tomatoes [35]. The high intake of glucose could result in toxicity of β-cells leading to high fasting glucose and MetS [36, 37]. Alternatively, high glucose intake could result in central obesity leading to a higher risk of MetS. However, the latter is unlikely to be the major mechanism since the observed association between vegetables and fruit intake with MetS was independent of adiposity.

Irrespective of our observations, we suggest that education on a healthy diet with the correct amount of vegetables and fruits with low glycemic indices, as well as other non-vegetable foods, might have a positive health effect in reducing MetS [38]. Further studies are warranted to understand how lifestyle changes could help reduce the MetS burden among PLHIV in Tanzania.

In the currency analysis, we analyzed fat mass index rather than body mass index (BMI) as a measure of adiposity because BMI includes both lean and fat mass and may attenuate the effect of adiposity on MetS. In this report, we found that, in a multivariable model, people in the upper tertile of the fat mass index had an increased risk of MetS. This is because increased body fat increases the risk of central obesity, insulin resistance, hyperglycemia, diabetes mellitus, atherogenesis, and the development of hypertension [39] which are the building blocks for MetS. Thus, incorporating body fat measurements in HIV clinics would early identify people at high risk of MetS and institute lifestyle interventions to reduce the risk of MetS and other non-communicable diseases.

The risk of MetS in the older age group has been observed to be high, these findings were expected and they are in line with the literature. Increased age has been documented to increase the risk of all MetS traits including fasting hyperglycemia, hypertension, and lipid abnormalities [40, 41]. Aging decreases the ability of pancreatic β-cells to manufacture and secrete insulin and increases the risk of diabetes [41], reduced vascular distensibility, and increases the risk of hypertension. Furthermore, aging increases adipose tissues dysfunction, increases the risk of lipid abnormalities, and increases the risk of cardiovascular disease [41, 42]. These findings suggest the need to prioritize interventions for the aging population to reduce their MetS and other non-communicable diseases risk.

## Strengths and limitations

The current study is among the few studies that have explored the prevalence of MetS in PLHIV. The study included PLHIV who are on old and new ART regimens making it possible to assess if new ART drugs might change the burden of MetS and thus making it easier to generalize the findings in the wider HIV population. However, the study had several limitations. It was a cross-sectional study so causal associations cannot be confirmed. Also, the switching of the older ARTs to dolutegravir a few years before this study may explain the lack of association that has been observed. Information on the ART duration was missing in a majority of participants. Only 35% had date of ART initiation recorded in their current ART card, proper recording of all information in ART cards should be emphasized. Physical activity data collected was reported rather than observed and the study may have been susceptible to social desirability bias although we validated instruments used in data collection.

## Conclusions and recommendations

This study concludes that, compared to the global prevalence of MetS, the prevalence of MetS in PLHIV is still high in Tanzania despite transitioning to newer and better ART regimens. We suggest routine identification of people with MetS to enhance the interventions done at treatment and care HIV clinics. We also found fat mass was associated with elevated MetS risk, fat mass screening would therefore benefit PLHIV, we suggest that this measurement should be included in HIV care to identify at-risk populations for early interventions and reduce the risk of MetS to improve long-term health among PLHIV.Using tools that were pre-tested and validated in other studies [33], we found adequate intake of vegetables and fruits increases MetS burden. This calls for longitudinal studies in PLHIV to re-examine the effect of vegetable/fruits intake in MetS in Tanzania.

## Electronic supplementary material

Below is the link to the electronic supplementary material.

Supplementary Material 1

## Data Availability

The data set used during the current study is available from the corresponding authors upon reasonable request.

## List of Abbreviations

HIV: Human immunodeficiency virus
PLHIV: People living with HIV
MetS: Metabolic syndrome
ART: Antiretroviral therapy
NCD: Non-communicable diseases PI:Protease inhibitors
NRTI: Nucleoside reverse transcriptase inhibitors
MET: Metabolic equivalents of tasks
LDL: Low-density lipoprotein
HDL: High-density lipoprotein
FMI: Fat mass index
IDF: International diabetes federation
WHO: World health organization
