# Supplementary figures and images for: Metabolic syndrome among people living with HIV on antiretroviral therapy in Mwanza, Tanzania
Source: BMC Endocr Disord. 2023 Apr 21;23:88. doi: 10.1186/s12902-023-01340-3 (PMC10120112; doi:10.1186/s12902-023-01340-3)

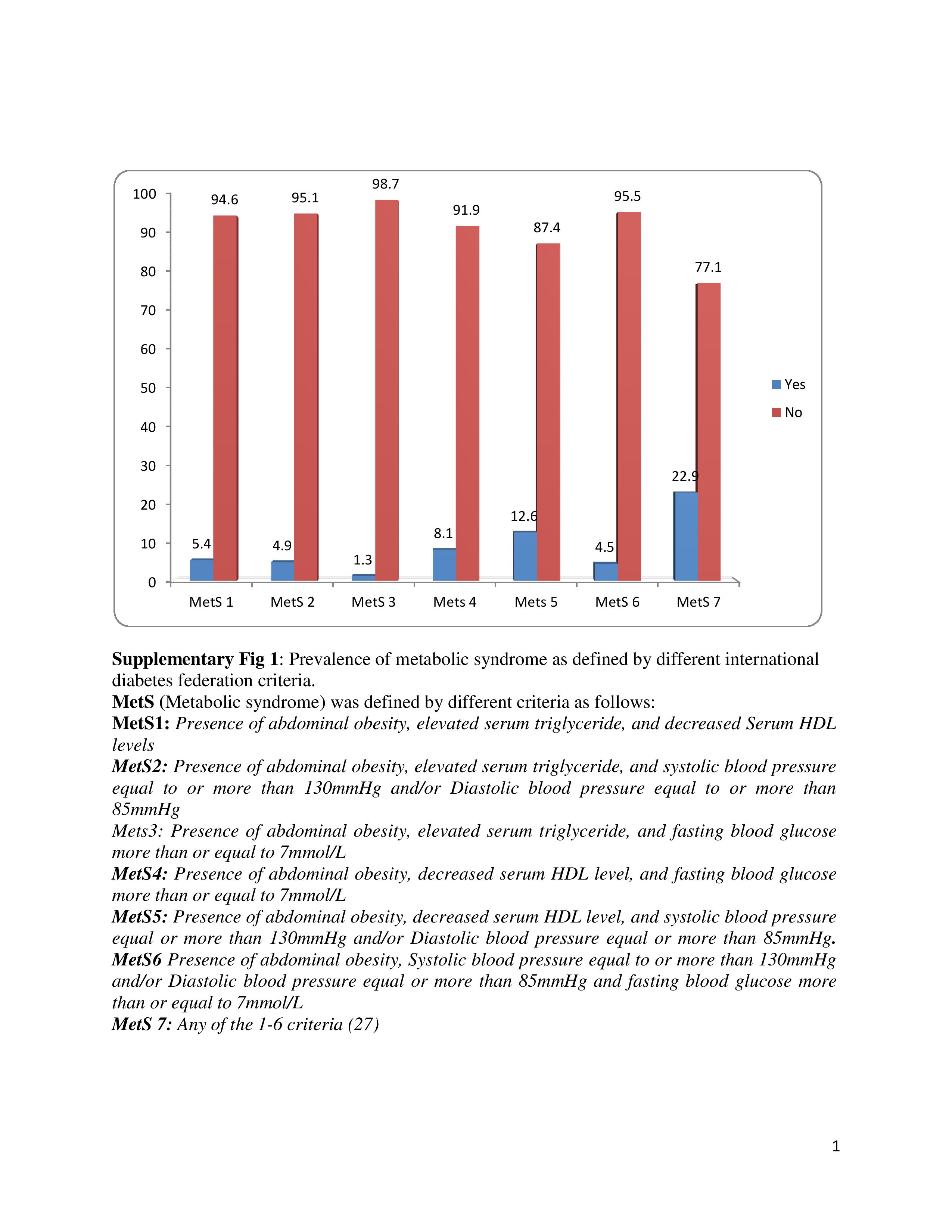

Supplement: Supplementary file 1 — Supplementary Material 1 [file 12902_2023_1340_MOESM1_ESM.jpg]
